# An Immune-Clinical Prognostic Index (ICPI) for Patients With *De Novo* Follicular Lymphoma Treated With R-CHOP/CHOP Chemotherapy

**DOI:** 10.3389/fonc.2021.708784

**Published:** 2021-07-13

**Authors:** Yaxiao Lu, Jingwei Yu, Wenchen Gong, Liping Su, Xiuhua Sun, Ou Bai, Hui Zhou, Xue Guan, Tingting Zhang, Lanfang Li, Lihua Qiu, Zhengzi Qian, Shiyong Zhou, Bin Meng, Xiubao Ren, Xianhuo Wang, Huilai Zhang

**Affiliations:** ^1^ Department of Lymphoma, Tianjin Medical University Cancer Institute and Hospital, National Clinical Research Center of Cancer, Key Laboratory of Cancer Prevention and Therapy, Sino-US Center for Lymphoma and Leukemia Research, Tianjin, China; ^2^ Departments of Pathology and Immunology/Biotherapy, Tianjin Medical University Cancer Institute and Hospital, Tianjin, China; ^3^ Department of Hematology, Shanxi Provincial Cancer Hospital, Taiyuan, China; ^4^ Department of Oncology, Second Hospital of Dalian Medical University, Dalian, China; ^5^ Department of Hematology, Cancer Center, The First Hospital of Jilin University, Changchun, China; ^6^ Department of Lymphoma & Hematology, Hunan Cancer Hospital, The Affiliated Cancer Hospital of Xiangya School of Medicine, Central South University, Changsha, China

**Keywords:** follicular lymphoma, peripheral blood, T lymphocyte subsets, prognostic index, risk stratification

## Abstract

**Purpose:**

Although the role of tumor-infiltrating T cells in follicular lymphoma (FL) has been reported previously, the prognostic value of peripheral blood T lymphocyte subsets has not been systematically assessed. Thus, we aim to incorporate T-cell subsets with clinical features to develop a predictive model of clinical outcome.

**Methods:**

We retrospectively screened a total of 1,008 patients, including 252 newly diagnosed *de novo* FL patients with available peripheral blood T lymphocyte subsets who were randomized to different sets (177 in the training set and 75 in the internal validation set). A nomogram and a novel immune-clinical prognostic index (ICPI) were established according to multivariate Cox regression analysis for progression-free survival (PFS). The concordance index (C-index), Akaike’s information criterion (AIC), and likelihood ratio chi-square were employed to compare the ICPI’s discriminatory capability and homogeneity to that of FLIPI, FLIPI2, and PRIMA-PI. Additional external validation was performed using a dataset (n = 157) from other four centers.

**Results:**

In the training set, multivariate analysis identified five independent prognostic factors (Stage III/IV disease, elevated lactate dehydrogenase (LDH), Hb <120g/L, CD4+ <30.7% and CD8+ >36.6%) for PFS. A novel ICPI was established according to the number of risk factors and stratify patients into 3 risk groups: high, intermediate, and low-risk with 4-5, 2-3, 0-1 risk factors respectively. The hazard ratios for patients in the high and intermediate-risk groups than those in the low-risk were 27.640 and 2.758. The ICPI could stratify patients into different risk groups both in the training set (P < 0.0001), internal validation set (P = 0.0039) and external validation set (P = 0.04). Moreover, in patients treated with RCHOP-like therapy, the ICPI was also predictive (P < 0.0001). In comparison to FLIPI, FLIPI2, and PRIMA-PI (C-index, 0.613-0.647), the ICPI offered adequate discrimination capability with C-index values of 0.679. Additionally, it exhibits good performance based on the lowest AIC and highest likelihood ratio chi-square score.

**Conclusions:**

The ICPI is a novel predictive model with improved prognostic performance for patients with *de novo* FL treated with R-CHOP/CHOP chemotherapy. It is capable to be used in routine practice and guides individualized precision therapy.

## Introduction

Follicular lymphoma (FL) is the second most common form of non-Hodgkin’s lymphoma and accounts for 20-30% of all adult lymphomas diagnosed worldwide (1, 2). It is an indolent lymphoma with heterogeneity both in treatment strategies and clinical outcomes (3, 4). The treatment approaches cover a range from a watch-and-wait strategy to CD20-directed immunotherapy alone or in combination with chemotherapy (5–7). Due to the importance of the immunological microenvironment in the oncogenesis and progression of FL, immunomodulatory drugs such as lenalidomide in combination with rituximab produced significant response rates in first-line therapy (8, 9). To stratify risk categories within FL patients, a variety of clinical prognostic models have been identified such as the Follicular Lymphoma International Prognostic Index (FLIPI) and FLIPI-2, and also proved as useful predictors of outcome (10, 11). Despite their frequent use, there are some distinct outcomes within risk groups, suggesting the need for additional prognostic parameters (7, 12). However, these models are not enough to reliably guide patient treatment options, while the GELF (Groupe d’Etude des Lymphomes Folliculaires) criteria (13) which includes parameters of tumor burden serves as an indication for initial treatment. Recently, several molecular biomarkers and gene signatures with prognostic significance have been discovered (14, 15). Several clinicogenomic models have been established to clarify the patients that are expected to exhibit poor outcomes after providing standard immunochemotherapy. For instance, the m7-FLIPI risk score, integrating the FLIPI with the Eastern Cooperative Oncology Group performance status (ECOG PS) and the mutation status of seven genes (EZH2, FOXO1, EP300, CREBBP, CARD11, MEF2B, ARID1A) appears to be better for the identification of high-risk patients compared with the existing clinical models (16, 17). Additionally, a developed 23-gene signature model could capture multiple aspects of the tumor biology and identify patients with markedly distinct outcomes (18). Molecular markers and gene signatures, on the other hand, are costly, technically difficult, and not routinely accessible in most hospitals. Recently, it was demonstrated that lack of expression of the intrafollicular CD4+ T-cell predicted risk of early failure, and by integrating this microenvironment biomarker with the FLIPI, termed BioFLIPI, which could further enhance the identification of FL patients at risk of early failure (19). Considering its complexity and poor reproducibility, it remains of great interest for us to investigate the impact of peripheral blood T cell subsets on patients’ clinical outcomes, which provides a simple indicator of the host’s immune status.

Tumor microenvironments have been recognized to be an imperative factor in the pathogenesis of follicular lymphoma (20, 21). Recently, several studies found that the composition of non-malignant cells, rather than tumor cells, influenced FL prognosis, emphasizing the modulation of the clinical course of follicular lymphoma by immune responses (22–25). Subsequently, various researches also tried to identify phenotypic markers to stratify patients, but this has proven to be more difficult than anticipated and also influenced by treatments (26–28). To the best of our knowledge, T lymphocytes can directly reflect the status of the body’s immune function and changes in the number of subsets of T lymphocytes had predictive value in disease progression. Further, its distribution can be different in peripheral blood and tumor microenvironment (29, 30). The T-cell subset can be used as a surrogate biomarker for the tumor microenvironment. Thus, this motivated us to identify the potential role of the peripheral blood T-cell subsets in FL, since its prognostic role was previously reported in diffuse large B-cell lymphoma (DLBCL) (31, 32).

Here, we have established an easily applicable prognostic nomogram and proposed a new prognostic model, termed the Immune Clinical Prognostic Index (ICPI) — based on peripheral blood T-lymphocytes (allowing ease of application in routine practice). This new model possesses the capability to risk-stratify patients well and predicts survival of the currently recommended regimen based on first-line immunochemotherapy. In comparison to traditional clinical scoring systems like FLIPI, FLIPI2 and PRIMA-PI, the performance of this suggested ICPI showed enhanced predictive abilities and it was convenient to be monitored dynamically in clinical application.

## Method

### Study Population

A total of 1,008 patients were retrospectively screened and reviewed by two experienced haematopathologists for diagnostic confirmation at Tianjin Medical University Cancer Institute and Hospital (TMUCIH) between January 2011 and July 2020 (**Figure S1**). We excluded the 737 patients (73%) for the following reasons: unavailability of peripheral blood T lymphocytes subsets (n = 630); histologically confirmed grade 3b FL (n=28); DLBCL with an additional FL 3b component (n=39); unavailability of other NHL or pathological data (n = 15); previously accepted chemotherapy (n = 25). The residual 271 histologically confirmed grade 1-3a *de novo* FL patients were further screened. Patients who were assigned to a wait-and-see (WW) policy (n = 7), refused to consider any therapy (n=4), received lenalidomide plus rituximab (n = 3), received single-agent rituximab (n = 2) or received fewer than two cycles of therapy (n=3) were not included. Finally, 252 patients with indications for treatment were found to be eligible for model construction and were randomly assigned to a training set (n = 177) and an internal validation set (n = 75) by 7:3. Overall, 141 (56%) patients met the GELF criteria. External validation was performed in an independent set (n = 157) from other four centers, including Shanxi Provincial Cancer Hospital, Second Hospital of Dalian Medical University, The First Hospital of Jilin University and Hunan Cancer Hospital, who were treated with RCHOP (rituximab,cyclophosphamide, doxorubicin, vincristine, and prednisone). The study was reviewed and approved by the Research Ethics Committee of the TMUCIH and other centers.

### Flow Cytometry Analysis of Peripheral Blood T-Cell Subsets

The cells were stained with the anti-CD3- fluorescein isothiocyante (FITC), anti-CD4-FITC, and anti-CD8- phycoerythrin (PE) monoclonal antibodies. After staining, the results were examined through Cell Quest software and FACSCalibur flow cytometer (Becton–Dickinson, USA). The percentage of lymphocytes positive for each antibody was identified as a result.

### Construction and Validation of the Nomogram

The least absolute shrinkage and selection operator (LASSO) is a widely used system for regression (33), which was used for the screening of independent variables that affect outcomes and to prevent overfitting. The “glmnet” package was used to generate this regression. To find independent prognostic factors for progression-free survival (PFS), researchers used multivariate Cox proportional hazards regression. The coefficient of regression of the selected independent variables was used to construct the corresponding nomogram prediction model. Internal validation was performed, and a C-index was calculated by investigating the area under the receiver operating characteristic curve. Calibration plots were used to evaluate whether the observed and predicted survival probabilities were in concordance with the bootstrap resampling method (B=1000). The “rms” package was used to draw the calibration curve and internal verification of the nomogram.

### Comparison Between Different Models

The ICPI score was evaluated by determining the risk categorization effect on PFS and which was then compared with the results obtained through developed prognostic indices (FLIPI, FLIPI-2 and PRIMA-PI). We compared the ICPI model with FLIPI, FLIPI2, and PRIMA-PI based on C-index and Akaike’s information criterion (AIC) (34–36). The AIC seems to have a relative model quality measure; a better model would be based on smaller values. As a general guide, AIC differences < 2 between models show no improved model fitting, differences > 2 but < 10 reveal an improved fit, and <10 indicates a significant improvement in model fitting (37). The C-index measured the model’s predictive capability, demonstrated as the possibility of concordance between observed and predicted survival. The C-index corresponds to the area under the receiver operating characteristics curve. The values of the C-index equivalent to 0.5, 0.7, and 1.0 represent that the random, acceptable, or perfect discrimination model, respectively, between long and short survival times. To measure homogeneity, the likelihood ratio chi-square was calculated using Cox regression; a higher likelihood ratio chi-square score indicated better homogeneity.

### Statistical Analysis

PFS was calculated from the initial treatment date to the date of death occur *via* any cause, disease progression or relapse, or last contact. The Kaplan-Meier method was used to estimate PFS, which was then compared among the groups with the help of a 2-sided log-rank test. The counts of T lymphocyte subsets were intended by using the percentages obtained by peripheral blood flow cytometry (PBFCM). Categorical variables were analyzed using the χ^2^ test or Fisher’s exact test. Using standard clinical thresholds, continuous biological variables were dichotomized. Especially, the determination of the optimal cut-off values for peripheral blood T lymphocyte subsets was accomplished by X-tile 3.6.1 software (Yale University, New Haven, CT, USA) (38) which was based on the highest value of χ^2^ defined by the log-rank test and Kaplan-Meier survival analysis.

Each statistical test was two-sided. Statistical significance was set at 0.05 and the analyses were accomplished with SPSS 25.0 (SPSS Inc. Chicago, IL) and R software (version 4.0.3; http://www.r-project.org/).

## Results

### Clinicopathological Characteristics of Patients

Patients’ characteristics were not significantly different between the training set and internal validation set (**Table 1**). A total of 252 newly diagnosed FL patients were examined, the median age was 52 years (range, 24–81), and most of them suffering from stage III-IV disease (83%). There were 72% of patients who had more than five involved lymph nodes, 17% showed the value of more than 6 cm for the longest diameter of the largest involved node (LoDLIN). Involvement of bone marrow was found in 20% of patients, and 31% showed spleen involvement. Hemoglobin (Hb) level below 120 g/L occurred in 19% and elevated lactate dehydrogenase (LDH) and β2-microglobulin (β2-MG) were found to be 15% and 28% of patients, respectively. Overall, 218 patients received CHOP plus rituximab (RCHOP)-like therapy and 34 patients received CHOP-like therapy.

**Table 1:** Baseline patient characteristics.

Characteristics	All patients	%	Training set	%	Internal validation set	%	P-value
	(n = 252)		(n = 177)		(n = 75)		
**Age**							0.768
≤60 years	178	71	126	71	52	69	
>60 years	74	29	51	29	23	31	
**Sex**							0.215
Male	126	50	93	53	33	44	
Female	126	50	84	47	42	56	
**Ann Arbor Stage**							
I/II	42	17	31	18	11	15	0.579
III/IV	210	83	146	82	64	85	
**B symptoms**							
Absence	212	84	147	83	65	87	0.473
Presence	40	16	30	17	10	13	
**Performance status (ECOG)**							
0-1	249	99	175	99	74	99	0.892
>1	3	1	2	1	1	1	
**Number of involved lymphonodes**							
<5	70	28	53	30	17	23	0.238
≥5	182	72	124	70	58	77	
**LoDLIN**							
≤6cm	202	80	144	81	58	78	0.256
>6cm	42	17	26	15	16	21	
Not available	8	3	7	4	1	1	
**Bone marrow involvement**							0.744
Absence	201	80	147	83	54	72	
Presence	51	20	30	17	21	28	
**Spleen involvement**							0.141
Absence	175	69	118	67	57	76	
Presence	77	31	59	33	18	24	
**Albumin <40 g/L**							0.563
Normal	207	82	147	83	60	80	
Decreased	45	18	30	17	15	20	
**LDH**							0.245
Normal	215	85	154	87	61	81	
Elevated	37	15	23	13	14	19	
**β-2 MG**							0.573
Normal	182	72	126	71	56	75	
Elevated	70	28	51	29	19	25	
**Hb(g/L)**							0.291
≥120	205	81	141	80	64	85	
<120	47	19	36	20	11	15	
**Platelets (10^9/L)**							0.581
≥150	203	81	141	80	62	83	
<150	49	19	36	20	13	17	
**CD4+**							
≥30.7%	168	67	116	66	52	69	0.559
<30.7%	84	33	61	34	23	31	
**CD8+**							
≤36.6%	193	77	137	77	56	75	0.639
>36.6%	59	23	40	23	19	25	
**CD4+/CD8+**							
≥0.8	198	79	136	77	62	83	0.302
<0.8	54	21	41	23	13	17	
**Rituximab-containing regimens**							
Yes	218	87	154	87	64	85	0.722
No	34	13	23	13	11	15	

ECOG, Eastern Cooperative Oncology Group; LoDLIN, longest diameter of the largest involved node; LDH, lactate dehydrogenase; β2-MG, β2-microglobulin; Hb, Hemoglobin.

Based on all three clinical risk rating systems, all patients were divided into categories. Especially, 8 patients’ LoDLIN could not be obtained so that FLIPI2 was evaluated among 244 patients. According to FLIPI, 23% patients were low risk, 41% patients were intermediate risk and 36% patients were high risk. In the proposed FLIPI2 score, specifically for the rituximab-treated patients, 65% patients were low risk, 20% patients were intermediate risk and 12% patients were high risk. In the PRIMA-PI scoring system, 66% patients were low risk, 9% patients were intermediate risk and 24% patients were high risk (**Table S1**).

### Cutoff Values of Peripheral Blood T Lymphocyte Subsets

**Figure 1:**
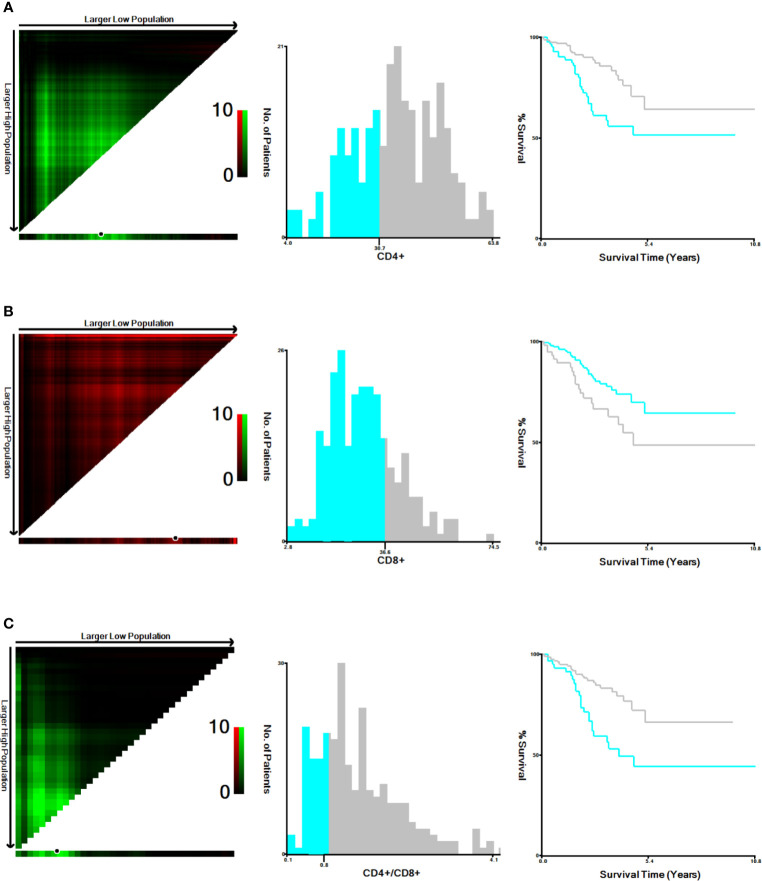
Cut-off values determination for CD4+, CD8+ and CD4+/CD8+ and survival analyses. The optimal cut-off values, denoted by black circles in the left panels, are displayed in histograms of the entire cohort (middle panels), and Kaplan-Meier plots are displayed in the right panels. **(A)** The optimal cut-off values for CD4+ was 30.7% (χ^2^ = 10.036, P=0.005). **(B)** for CD8+ was 36.6% (χ^2^ = 5.238, P =0.023). **(C)** for CD4+/CD8+ was 0.8 (χ^2^ = 10.637, P=0.006).

In our study, the cut-off values of CD4+ T cells, CD8+ T cells levels and CD4+/CD8+ ratio were determined by X-tile program, which were found to be 30.7%, 36.6% and 0.8, respectively (**Figure 1**). The χ^2^ log-rank value of CD4+, CD8+ and CD4+/CD8+ were 10.036, 5.238 and 10.637, respectively. To conduct further study, the patients were distributed into 2 groups (CD4+ <30.7% and ≥30.7%, CD8+ ≤36.6% and >36.6%, CD4+/CD8+ <0.8 and ≥0.8). Overall, 84 (33%) patients presented lower CD4+ T-cell percentage and 59 (23%) showed higher percentage of CD8+ T-cell. A total of 54 (21%) of patients showed CD4+/CD8+ ratio below 0.8 (**Table 1**).

### Clinical Value of the Nomogram

**Figure 2:**
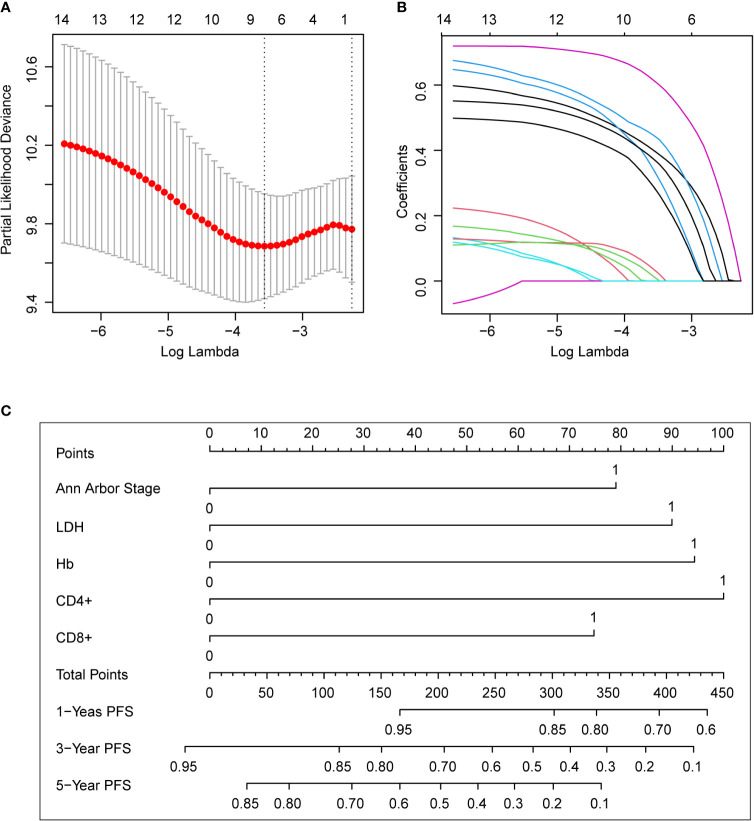
The predictive factors were selected using LASSO regression analysis and nomogram construction. **(A)** Screening for tuning parameter (lambda) in the LASSO regression model. The partial likelihood deviance was calculated as a function of log (lambda), with the least deviance in partial probability corresponding to the optimal number of variables. The dotted vertical lines represented the optimal lambda value on the basis of 1 standard error and the minimum criteria. **(B)** The profiles of LASSO coefficient of the non-zero variables of FL patients. When 8 variables remained, the lowest partial probability deviance was observed. **(C)** For using the nomogram, place the value assigned to the individual patient on each variable axis, and draw an upward line for the determination of the number of points received for each variable value. The sum of these numbers is obtained on the total points axis, and a line is drawn down to the survival axis to calculate the 1, 3, 5-year PFS likelihood. LDH, lactate dehydrogenase; PFS, progression-free survival.

In the training set, we used the Lasso regression approach to distinguish adverse prognostic factors due to the limited sample size and a large number of variables. The cross-validation and filtering practices of the independent variables are given in **Figures 2A, B**, respectively. Lambda. min identified the superb performance model with the least number of independent variables. LASSO regression analysis included the fourteen variables. The variables were age, Ann Arbor stage, sex, number of involved nodal sites, bone marrow involvement, spleen involvement, LDH, β2-MG, hemoglobin, platelet counts, serum albumin, and peripheral blood T lymphocyte subsets such as CD8+ T cells, CD4+ T cells, and CD4+/CD8+ ratio. Finally, eight potential variables of the 177 individuals in the training set were prognostic factors when the partial likelihood deviance was the smallest, which were as follows: age, stage III/IV disease, elevated LDH, Albumin <40 g/L, Hb <120g/L, CD4+ <30.7%, CD8+ >36.6% and CD4+/CD8+ <0.8. Subsequently, we performed multivariate analyses of the obtained 8 factors, and the results revealed that Ann Arbor stage, Hb, LDH, CD4+, and CD8+ were independent predictors for PFS (**Table S2**).

**Figure 3:**
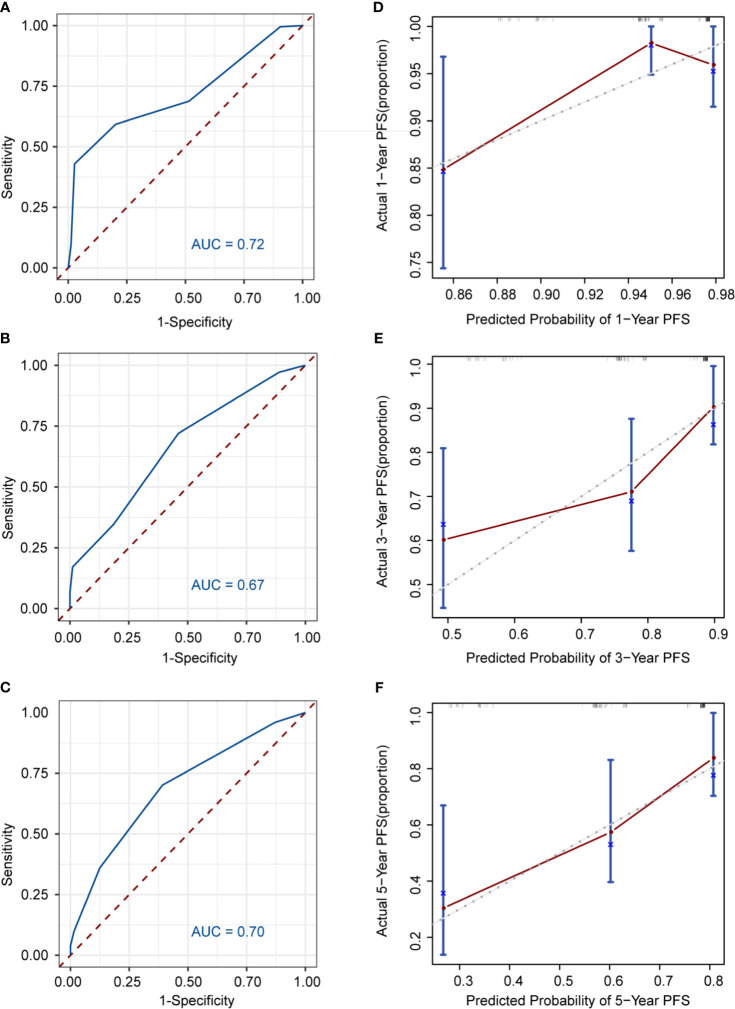
Discrimination and calibration of the nomogram to predict 1, 3, 5-year PFS likelihoods in patients with follicular lymphoma. The area under the receiver operating characteristic (ROC) curve (AUC) and the calibration curve for the prediction of 1-year PFS **(A, B)**, 3-year PFS **(C, D)**, 5-year PFS **(E, F)**; The PFS probability predicted by, the nomogram is plotted on x-axis; while the actual PFS is plotted on the y axis.

Nomogram was developed to predict 1, 3, 5-year PFS upon the multivariate analysis results (**Figure 2C**). Variables included stage III/IV disease, elevated LDH, Hb <120g/L, CD4+ <30.7%, CD8+ >36.6% entered the nomogram. The predictive accuracy for 1, 3, 5-year C-index measured PFS was 0.72, 0.67 and 0.70 respectively (**Figures 3A–C**). The calibration curve for the likelihood of 1, 3, and 5-year PFS revealed a strong connection between the observed result and the nomogram’s prediction. (**Figures 3D–F**). **Figure S2** shows the 1, 3, and 5-year survival calibration curves of the internal validation and external validation sets, which are similar to that of the training set.

### Establishment of ICPI and Comparison With Conventional Models

To assist the applicability of the predictive model, we developed a novel ICPI based on the five risk factors (Ann Arbor stage III/IV disease, elevated LDH, Hb < 120g/L, CD4+ < 30.7%, CD8+ > 36.6%). Patients were distributed into three risk categories based on the number of risk factors they offered: low (0-1 risk factor), intermediate (2-3 risk factors), and high (4-5 risk factors). The risk ratios for patients belonging to the intermediate and high-risk groups versus those present in the low-risk group were 2.758 (95% CI: 1.182-6.433) and 27.640 (95% CI: 8.606-40.843), respectively (**Table S3**). Patients repartition in each ICPI risk group in the training set were detailed in **Table S4**. When comparing ICPI with FLIPI, 41% of patients were in the same risk group, whereas 54% were in adjacent risk categories. Risk classifications were largely different in 8 patients (5%; 8 patients were classified as low risk by ICPI but high risk by FLIPI). As for ICPI and FLIPI2, 57% of patients were in the same risk category using ICPI and FLIPI2, whereas 41% were in adjacent risk categories. Risk classifications were largely different in 3 patients (2%; 2 patients were classified as low risk by ICPI but high risk by FLIPI2, and 1 patient was classified as high risk by ICPI but low risk by FLIPI2). To compare the ICPI with PRIMA-PI, 47% of patients were in the same risk category using ICPI and PRIMA-PI, whereas 42% were in adjacent risk categories. Risk classifications were largely different in 18 patients (10%; 14 patients were classified as low risk by ICPI but high risk by PRIMA-PI and 4 patient was classified as high risk by ICPI but low risk by PRIMA-PI).

**Figure 4:**
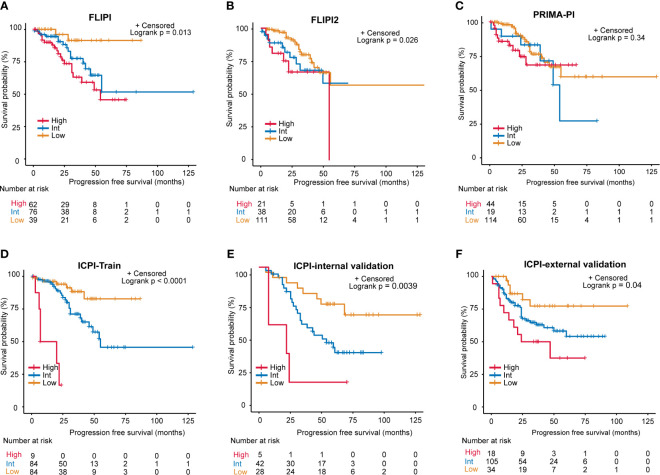
Progression-free survival (PFS) for risk groups defined by 4 scoring systems. **(A)** FLIPI, **(B)** FLIPI2, **(C)** PRIMA-PI, **(D)** Training set of the ICPI, **(E)** Internal validation set of the ICPI, **(F)** External validation set of the ICPI.

We then conducted Kaplan-Meier curves to estimate the effect of different risk groups on patients’ PFS time according to the ICPI, FLIPI, FLIPI2 and PRIMAPI (**Figures 4A–D**). All of the scores could differentiate subgroups of patients with substantially different prognoses except for the PRIMA-PI (P = 0.34). The ICPI (P<0.0001) is comparable to FLIPI (P=0.013) in stratifying patients into different risk groups. However, the FLIPI2 and PRIMA-PI were unsatisfactory for stratifying patients between intermediate and high-risk groups. According to ICPI, 84 (47.5%) patients were classified at low risk, 84 (47.5%) were intermediate risk, and 9 (5%) were high risk. Two-year PFS was 94% (95% CI, 88%-100%), 95% (95% CI, 90%-99%), and 33% (95% CI, 12%-96%) for the ICPI in the low, intermediate and high risk groups, respectively. In both the internal validation (P = 0.0039) and external validation sets (P = 0.04), the ICPI could also classify patient into different risk groups (**Figures 4E, F**). In the external validation sets according to ICPI, 34 patients (22%) were classified at low risk, 105 (67%) at intermediate risk, and 18 (11%) at high risk. Additionally, in our training set of patients treated with RCHOP-like therapy (49%, 47%, and 4% for patients at low risk, intermediate risk, and high risk, respectively; P < 0.0001), the ICPI was also predictive (**Figure S3**). Therefore, the ICPI proved to be a robust tool for predicting survival.

As for model performance, when compared with FLIPI, FLIPI2, and PRIMA-PI, the ICPI provided the best data fitting designated by the lowest value of AIC (training set: ICPI *vs*. FLIPI **vs**. FLIPI2 *vs*. PRIMA-PI = 297 *vs* 312 *vs* 303 *vs* 320; internal validation set: ICPI *vs*. FLIPI *vs*. FLIPI2 *vs*. PRIMA-PI = 271 *vs* 272 *vs* 277 *vs* 280 **Table 2**). The ICPI also distinguished between patients with favorable and poor PFS (specified by the highest C-index: training set: 0.679, 0.647, 0.645, and 0.613, respectively, for models with ICPI, FLIPI, FLIPI2, and PRIMA-PI risk categories; internal validation set: 0.636, 0.619, 0.562, 0.507) (**Table 2**). Moreover, the ICPI had better performance based on the likelihood ratio chi-square (likelihood ratio chi-square, training set: ICPI = 25.29, FLIPI = 10.33, FLIPI2 = 5.79, PRIMA-PI = 2.05; internal validation set: ICPI = 9.31, FLIPI = 8.36, FLIPI2 = 4.60, PRIMA-PI = 0.81).

**Table 2:** Comparisons between model performance for PFS.

	Training set (n = 177)			
	ICPI	FLIPI	FLIPI2	PRIMA-PI
C-index† (95% CI)	0.679 (0.580-0.780)	0.647 (0.598-0.762)	0.645 (0.586-0.774)	0.613 (0.586-0.774)
AIC*	297.474	312.430	303.724	320.712
Likelihood ratio chi-square‡	25.29	10.33	5.79	2.05
	**Internal-validation set (n = 75)**			
	**ICPI**	**FLIPI**	**FLIPI2**	** PRIMA-PI**
C-index† (95% CI)	0.636 (0.592-0.768)	0.619 (0.594-0.766)	0.562 (0.582-0.778)	0.507 (0.588-0.772)
AIC*	271.584	272.538	277.735	280.086
Likelihood ratio chi-square‡	9.31	8.36	4.60	0.81

*The AIC provided a relative measure of the quality of the model; lower values indicate a better model fitting. AIC differences of < 2 designate no progress in fit, differences of > 2 but < 10 exhibited increasing progress in fit, and differences of more than 10 indicate significant improvement in model fit.

^†^The C-index provided a measure of the model’s predictive ability, defined as the likelihood of concordance between observed and predicted survival. The C-index corresponds to the area under the receiver operating characteristics curve. C-index values of 0.5, 0.7, and 1.0 suggest that the model discriminates between short and long survival periods in a random, permissible, or ideal manner, respectively.

^‡^A higher likelihood ratio chi-square score indicates better homogeneity.

## Discussion

To date, several prognostic indexes such as FLIPI and FLIPI2 are available to predict the prognosis in previously untreated FL patients; however, immunity of the host and tumor microenvironment is not considered by either FLIPI or FLIPI-2. The FLIPI was well-established to predict the OS *via* a retrospective study, and the FLPI2 was well-established to predict the PFS as the primary efficacy endpoint for model building (10, 11). However, they are not capable to accurately predict the early progress of patients (eg, POD24) after receiving first-line immunochemotherapy (39) and unable to provide evidence of risk-adapted treatment strategy. Furthermore, clinicogenomic models integrating genomic data into a clinical model, such as m7-FLIPI and 23-gene signature, may be difficult to implement because of cost and complexity. Therefore, the ability to recognize those high-risk patients who might develop progression or recurrence before treatment is still important because they can be candidates for alternative intensified therapies. Here, with the help of the obtained clinical features and laboratory indexes that are part of standard procedures used in the diagnosis, we have developed a predictor model of PFS based on peripheral blood T-lymphocytes and clinical features that stratify patients into different risk groups. The model we developed is based on immune-relevant characteristics of human bodies rather than recognized clinical prognostic factors.

It is well known that the status of the host immune is closely related to the development and occurrence of follicular lymphoma. T lymphocyte subsets including CD3+, CD4+, and CD8+ are the major components of the cellular immune system playing a leading role in antitumor immunity. Identifying the number of CD4+ T cells, CD8+ T cells, and the CD4+/CD8+ ratio in peripheral blood can thus represent the immunological state of patients with malignant tumors, and it may also be useful in predicting the prognosis of FL patients. The prognostic value of peripheral blood T-lymphocytes has also been reported in patients suffering from various subtypes of lymphoma (32, 40–42). However, no study has yet precisely reported the prognostic value of the T-cell subset in FL and its added value to clinical prognostic indices that identify patients especially at high risk of early death and progression even after the application of modern immunochemotherapy.

FL is a heterogeneous disorder, and over the last decade, a number of clinical parameters have emerged as significant prognostic factors (43, 44). Aiming to establish a prognostic system, we utilized variables of clinical significance that had been previously related to outcomes for FL, as well as peripheral blood T-lymphocytes which were immunologically relevant effective and simple biomarkers. Using the Cox Regression Multivariate Analysis, five variables (Ann Arbor Stage, Hb, LDH, CD4+, CD8+) have been identified as the most significant parameters affecting the survival of FL patients, and all these variables are easily obtainable at the time of diagnosis. Additionally, we established a nomogram to assess the probability of 1, 3, 5-year PFS, which could provide the risk assessment for each patient. The results showed that the nomogram possesses a good accuracy level for the prediction of PFS.

To our knowledge, this is the first research to combine clinical features with peripheral blood T-lymphocytes to predict outcomes in FL. Based on the risk factors offered by patients, we developed a new immune clinical prognostic index (ICPI). This risk score is feasible since it is based on features that are readily available at the time of diagnosis. Unlike existing prognostic scoring systems, ICPI could better stratify patients into different groups and revealed an upgraded ability for predicting PFS in comparison with PRIMA-PI or FLIPI-2, and a comparable ability to FLIPI. Most importantly, peripheral blood T-lymphocytes are simple to attain in clinical routine, and their complex changes over time can have significant clinical implications. In comparison to traditional prognostic models, the ICPI’s C-index for PFS prediction was superior to the predictive power of the FLIPI, FLIPI2, and PRIMA-PI. The key benefit of ICPI is that it combined peripheral blood T-lymphocyte subsets with traditional prognostic factors, which was found to stratify patients more efficiently than models that only used traditional prognostic factors, according to PFS. Hence, the model may be useful to identify the candidates for further immune-related research and clinical trial of the novel anti-immune drug. Additionally, although the ICPI helps to well stratify patients after the first-line treatment with rituximab plus chemotherapy, its prognosis value needs to be further explored among patients who received other frontline treatment like lenalidomide plus rituximab (R2), bendamustine and rituximab (BR) and single-agent rituximab in a large cohort study.

In conclusion, when compared to other models, the ICPI proposed in this study integrates peripheral blood T-lymphocytes, which represent the immune status of the host, and clinical risk factors and performs well in stratifying patients into distinct outcomes. This predictor is suitable to be used in routine practice and able to better adjust the existing options of therapy according to the patient risk category.

## Data Availability Statement

The original contributions presented in the study are included in the article/**Supplementary Material**. Further inquiries can be directed to the corresponding authors.

## Ethics Statement

The study was reviewed and approved by the Research Ethics Committee of the Tianjin Medical University Cancer Institute and Hospital and other four centers. The patients/participants provided their written informed consent to participate in this study.

## Author Contributions

HZha, XW, and SZ contributed to conception and design of the study. LS, XS, OB and HZha provided external validation cohort data. XG, TZ and BM performed the research and data analysis. YL wrote the first draft of the manuscript. JY, WG wrote sections of the manuscript. LL, LQ, and ZQ were responsible for collecting clinical information. XR supported the essential reagents. All authors listed have made a substantial, direct, and intellectual contribution to the work and approved it for publication.

## Funding

This work was supported by the National Natural Science Foundation of China (grant NO. 81770213), Natural Science Foundation of Tianjin (grants NO. 19JCYBJC26500 and 18JCZDJC45100), Clinical Oncology Research Fund of CSCO (grants NO. Y-XD2019-162, Y-Roche20192-0097), National Key New Drug Creation Special Programs (grant NO. 2018ZX09201015), and National Human Genetic Resources Sharing Service Platform/Cancer Biobank of Tianjin Medical University Cancer Institute and Hospital (grant NO. 2005DKA21300).

## Conflict of Interest

The authors declare that the research was conducted in the absence of any commercial or financial relationships that could be construed as a potential conflict of interest.
