# Supplementary material for: An Immune-Clinical Prognostic Index (ICPI) for Patients With De Novo Follicular Lymphoma Treated With R-CHOP/CHOP Chemotherapy
Source: Front Oncol. 2021 Jul 13;11:708784. doi: 10.3389/fonc.2021.708784 (PMC8316046; doi:10.3389/fonc.2021.708784)
Supplement: Supplementary file 1 [file DataSheet_1.docx]

**Supplementary Figures:**


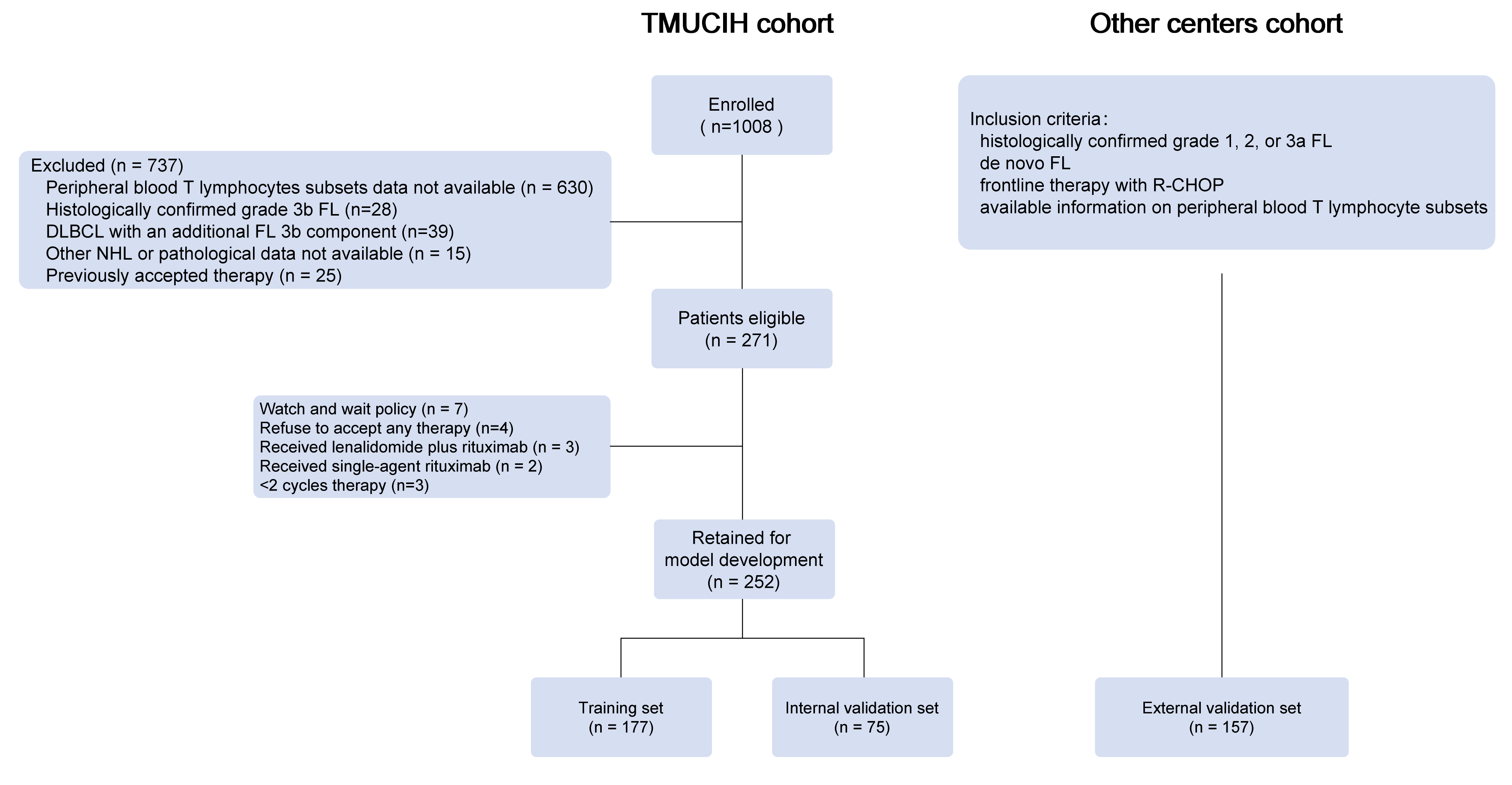


**Figure S1.** Flow chart of patients included in the analysis.


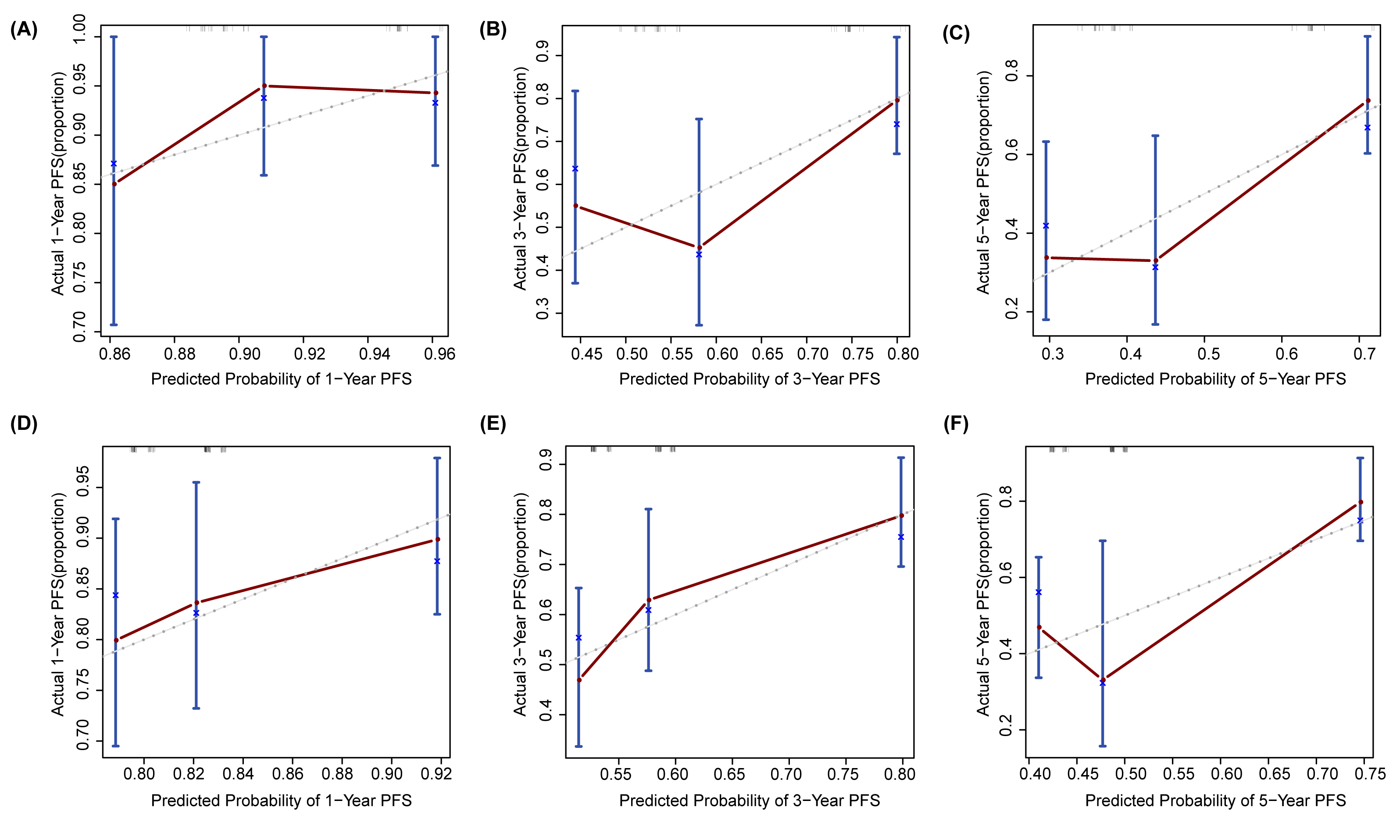


**Figure S2.** The calibration curves for the prediction of 1, 3, 5-year PFS in the internal validation set (A-C) and external validation set (D-F).

**
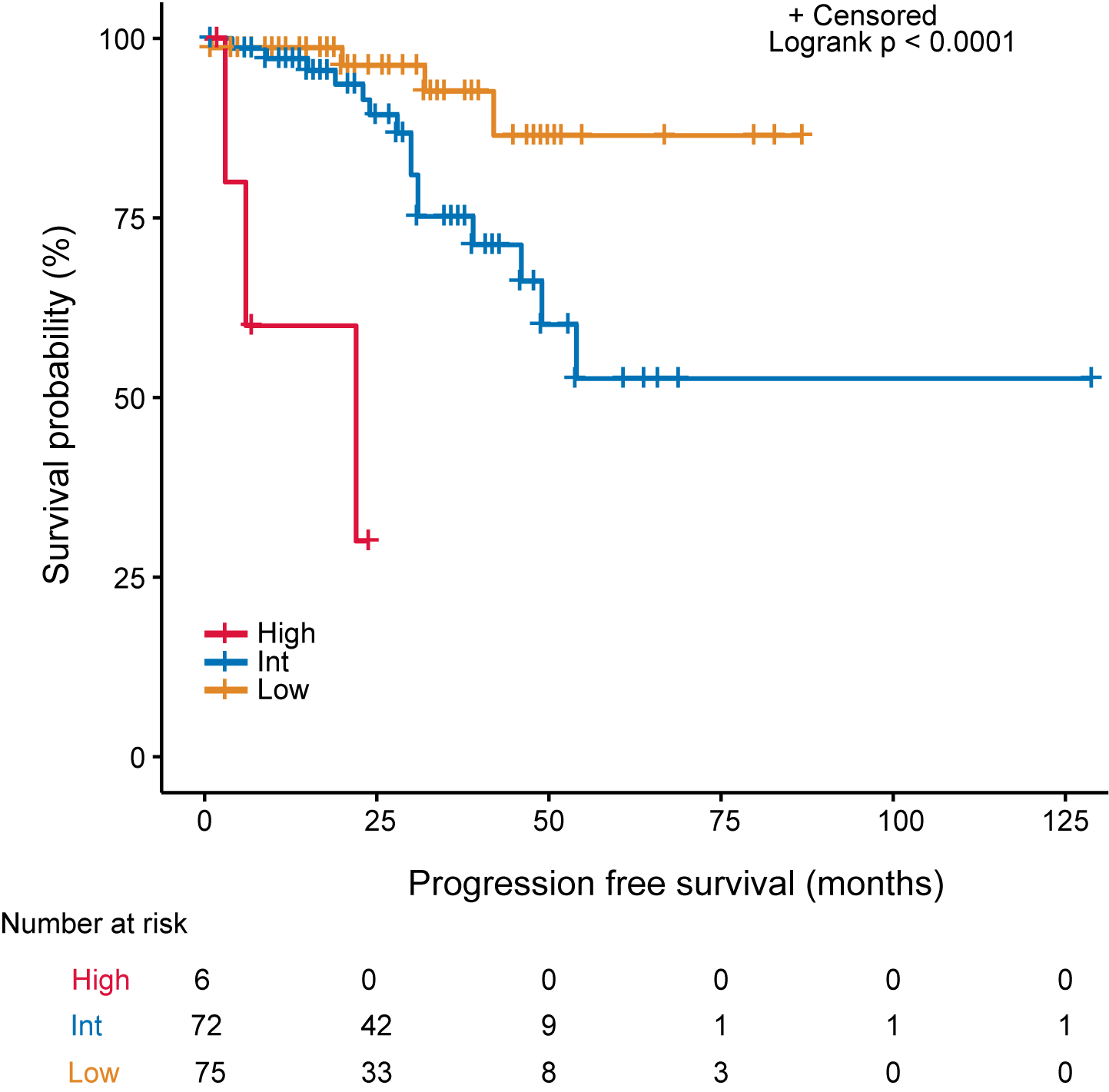
**

**Figure S3.** Progression-free survival (PFS) for ICPI risk groups in in the group of patients treated with RCHOP-like therapy.

**Supplementary Tables:**

**Table S1**. Traditional prognostic systems of patients with follicular lymphoma.

| **Prognostic systems** | **All patients** | **%** | **Training set** | **%** | **Internal  validation set** | **%** | **P-value** |
| --- | --- | --- | --- | --- | --- | --- | --- |
|  | **(n = 252)** |  | **(n = 177)** |  | **(n = 75)** |  |  |
| **FLIPI** |  |  |  |  |  |  | 0.377 |
| Low | 58 | 23 | 39 | 22 | 19 | 26 |  |
| Intermediate | 104 | 41 | 76 | 43 | 28 | 37 |  |
| High | 90 | 36 | 62 | 35 | 28 | 37 |  |
| **FLIPI-2** |  |  |  |  |  |  |  |
| Low | 164 | 65 | 111 | 63 | 53 | 71 | 0.152 |
| Intermediate | 50 | 20 | 38 | 21 | 12 | 16 |  |
| High | 30 | 12 | 21 | 12 | 9 | 12 |  |
| Not available | 8 | 3 | 7 | 4 | 1 | 1 |  |
| **PRIM-PI** |  |  |  |  |  |  | 0.226 |
| Low | 167 | 66 | 114 | 64 | 53 | 71 |  |
| Intermediate | 25 | 9 | 19 | 11 | 6 | 8 |  |
| High | 60 | 24 | 44 | 25 | 16 | 21 |  |

Abbreviations: FLIPI, Follicular Lymphoma International Prognostic Index; PRIMA-PI (PRIMA-prognostic index)

**Table S2**. Multivariate Analysis of PFS.

| **Parameters** | **Adverse factor** | **HR** | **95% CI** | **P** |
| --- | --- | --- | --- | --- |
| Ann Arbor Stage | III/IV | 2.005 | 1.012-3.797 | 0.048 |
| LDH | Elevated | 2.162 | 1.007-4.644 | 0.048 |
| Hb(g/L) | < 120 g/L | 2.231 | 1.101-4.520 | 0.026 |
| CD4+ | < 30.7% | 2.337 | 1.185-4.611 | 0.014 |
| CD8+ | > 36.6% | 1.905 | 1.022-3.766 | 0.042 |

Abbreviations: LDH, lactate dehydrogenase; Hb, Hemoglobin, HR, hazard ratio.

| **Risk group** | **Number of factors** | **HR** | **95% CI** |
| --- | --- | --- | --- |
| Low | 0-1 | 1.00 | － |
| Intermediate | 2-3 | 2.758 | 1.182-6.433 |
| High | 4-5 | 27.640 | 8.606-40.843 |

Table S3. Outcome and hazard risk of PFS according to risk group as defined by the Immune-Clinical Prognostic Index (ICPI).

Abbreviations: PFS, progression-free survival; HR, hazard ratio.

Table S4. Patients repartition in each ICPI risk group in the training set (N=177).

|  | **Risk group** | **FLIPI** | **FLIPI2** | **PRIMAPI** |
| --- | --- | --- | --- | --- |
| Low risk ICPI | Low | 32 | 69 | 65 |
| (N=84) | Intermediate | 44 | 10 | 5 |
|  | High | 8 | 2 | 14 |
|  | Not available | － | 3 | － |
| Intermediate risk ICPI (N=84) | Low | 7 | 41 | 45 |
|  | Intermediate | 32 | 24 | 14 |
|  | High | 45 | 15 | 25 |
|  | Not available | － | 4 | － |
| High risk ICPI | Low | 0 | 1 | 4 |
| (N=9) | Intermediate | 0 | 4 | 0 |
|  | High | 9 | 4 | 5 |
|  | Not available | － | － | － |
